# Decisions for Others Are Less Risk-Averse in the Gain Frame and Less Risk-Seeking in the Loss Frame Than Decisions for the Self

**DOI:** 10.3389/fpsyg.2017.01601

**Published:** 2017-09-15

**Authors:** Xiangyi Zhang, Yi Liu, Xiyou Chen, Xuesong Shang, Yongfang Liu

**Affiliations:** ^1^School of Psychology and Cognitive Science, East China Normal University Shanghai, China; ^2^Changsha Experimental Middle School Changsha, China; ^3^Key Laboratory of Brain Functional Genomics, Ministry of Education, Shanghai Key Laboratory of Brain Functional Genomics, East China Normal University Shanghai, China

**Keywords:** social distance, gain, loss, risk preference, loss aversion

## Abstract

Despite the fact that people make decisions for others as often as they make decisions for themselves, little is known about how decisions for others are different from those made for the self. In two experiments, we investigated the effect of social distance (i.e., making decisions for oneself, a friend, or a stranger) on risk preferences in both gain and loss situations. We found that people were more risk averse in gain situations when they made decisions for themselves than for a stranger (Studies 1 and 2), but were equally risk averse for themselves and their friends (Study 2). However, people were more risk seeking in loss situations when they made decisions for themselves than for their friends as well as for a stranger, and were more risk seeking for their friends than for a stranger (Study 2). Furthermore, the effect of social distance on risk preferences was stronger in loss than in gain situations. Mediation analysis indicated that outcome-induced loss aversion was responsible for effects of social distance on risk preferences. These findings demonstrate that social distance influences risk preferences via perceived loss aversion, which sheds new light on self-other differences in decision making.

## Introduction

Individual decisions are dramatically susceptible to the frame in which decision-making problems are described. The frame in terms of gains and losses has a remarkable influence on individual decision-making: When people make a choice between a risky and a sure option that have equal expected values, they tend to prefer the risky option in the loss frame, whereas they tend to be risk-averse in the gain frame (Kahneman and Tversky, 1979, 1984; Tversky and Kahneman, 1981).

However, risk preference can change for both types of frame. Research has identified several factors in determining such preference, including task type (Kühberger and Tanner, 2010), affect intensity (Zhang et al., 2016), and decision-maker personality traits such as sensation seeking (Benjamin and Robbins, 2007). Does it also make a difference whether decisions are made for socially distant others, socially close others, or for oneself? Specifically, will people be less risk-averse in the gain frame and less risk-seeking in the loss frame when they make decisions for a stranger than for a close friend or for themselves? The effect of social distance on risk preference is not well understood for either frame type. Therefore, we investigated how decisions for oneself or another person (i.e., social distance) can influence individuals’ risk preferences in both gain and loss frames (i.e., decision situations).

### Risk Preferences in Gain and Loss Frames

According to prospect theory, people are risk averse in the gain frame, preferring a sure gain to a speculative gamble, but are risk seeking in the loss frame, tending to choose a risky gamble rather than a sure loss (Kahneman and Tversky, 1979, 1984; Tversky and Kahneman, 1981). For example, when people face a choice between ‘a sure gain of $250’ and ‘a 25% chance to gain $1000,’ they tend to choose the former option. In contrast, when people face a choice between ‘a sure loss of $750’ and ‘a probability of 75% of losing $1000,’ they prefer the latter option.

Recently, a small research stream has documented individuals’ risk preferences in the gain and loss frames (Raue et al., 2015; Ziegler and Tunney, 2015; Sun et al., 2016). For example, using social, spatial, and temporal distance to represent psychological distance, Raue et al. (2015) investigated undergraduate students’ decisions in psychologically proximal conditions (e.g., imaging being a student, a new flu virus is currently threatening your city) and in psychologically distal conditions (e.g., imaging being a consultant in health care, some countries in the coming months will be threatened by an unusual disease) in both gain (a sure number of people will be saved and a probability that a larger number of people will be saved) and loss frames (a sure number of people will die and a probability that a larger number of people will die). They found that participants were more risk averse in the gain frame when decisions were made in psychologically proximal than in more distal conditions; however, participants were equally risk seeking in the loss frame. Similarly, Sun et al. (2016) asked the participants to make choices for themselves or for another person (an average student on their campus) during a poker game where participants could end this game to gain (or lose) a sure amount of money, or could continue to play leading to a probability of a larger gain (or loss). They showed that participants were more risk averse in the gain frame when decisions were made for themselves than for others, whereas no significant difference was observed between the two conditions in the loss frame.

### Loss Aversion and Risk Preferences

Loss aversion reflects a prevalent avoidance behavior involving choices that could result in losses (De Martino et al., 2010). Loss aversion demonstrates the fact that people are more sensitive to losses than to gains of the same magnitude. Losses are weighted roughly twice as strongly as gains (Tversky and Kahneman, 1992). For example, people typically avoid a gamble with an equal probability of either gaining $30 or losing $20, unless the amount of gain is roughly twice the amount of loss, that is, a 50% possibility of gaining $40 or losing $20.

Loss aversion has been used to account for framing effects on risk preference. Specifically, people are more afraid of the potential losses derived from a risky prospect in the gain frame, which contributes to the prevalence of risk aversion in choices between probable and sure gains. On the other hand, people are also more aversive to certain losses derived from a sure (riskless) prospect in the loss frame, which contributes to the prevalence of risk seeking in choices between probable and sure losses (Tversky and Kahneman, 1981, 1992). Some research also provides evidence in favor of the notion that risk preferences in both frames may be driven by loss aversion (Barkley-Levenson et al., 2013; Yechiam and Telpaz, 2013; Rieger et al., 2015). For example, Barkley-Levenson et al. (2013) demonstrated that participants tend to be risk-avoidant due to loss aversion when facing potential gains. Yechiam and Telpaz (2013) showed that participants tend to be more risk-seeking when the decision-making tasks involved potential losses, and they believed that this could be due to people paying more attention to losses.

Therefore, a sure option is preferred to a risky option in a gain frame and a risky alternative is preferred in a loss frame if people feel a strong sense of loss aversion (Ert and Erev, 2013). The stronger the level of loss aversion induced by a risky prospect in the gain frame and by a sure prospect in the loss frame, the more risk averse over gains and more risk seeking over losses people are.

### Social Distance and Loss Aversion

In daily life, individuals make decisions not only for themselves but also for others (Polman, 2010, 2012a; Stone et al., 2013). Furthermore, decisions for others depend heavily on the social distance between ourselves and others (Trope and Liberman, 2010; Sun et al., 2016). Social distance describes the affective closeness between others and ourselves, with a reference point of the self and a target being more psychologically removed from that point as social distance increases (for a review, see Trope and Liberman, 2010).

Previous studies have demonstrated that increased social distance works to reduce loss aversion. For example, Polman (2012b) investigated the impact of making decisions for the self and others on loss aversion across a range of varied contexts, involving exchanging gift cards (Study 1), playing coin-toss gambles (Study 2), playing or rejecting some lotteries (Study 3), and paying to improve or worsen social aspects of life (Studies 4a–e). They showed that loss aversion was markedly reduced when making decisions for others. Similarly, Mengarelli et al. (2014) explored participants’ choices for themselves and others across three economic tasks: Choosing between a sure option and a risky option of equal expected value (in task 1), pinpointing the minimum amount of gain accepted to play a gamble with a 50/50 chance to either gain or lose (in task 2), and choosing between two mixed gambles with a 50% chance to either gain or lose (in task 3). They found that loss aversion was remarkably mitigated when making decisions for others as compared with for the self. Additionally, using a virtual lab approach, Andersson et al. (2014) asked participants to make choices in four conditions (decisions for self with/without payment, both the decision maker and the receiver are paid, and decisions for others) that differed by whether they include the possibility of incurring losses. They also showed that decisions for others reduce loss aversion.

In sum, social distance between decision makers and targets influences loss aversion during the decision-making process (Polman, 2012b; Andersson et al., 2014). Decision makers show less loss aversion as social distance increases (i.e., from themselves to a stranger).

### Present Research

Few studies have investigated the effect of social distance on risk preferences in both gain and loss situations. Furthermore, the three existing studies (Raue et al., 2015; Ziegler and Tunney, 2015; Sun et al., 2016) used an imaginary or abstract target to indicate others. For example, Sun et al. (2016) used the designation ‘an average student on their campus’ to represent the “other.” However, when a decision is made for an abstract “other,” individuals would be emotionally more distant from the “other,” which leads to more difficulty imagining how the “other” feels about risk (Hsee and Weber, 1997). In addition, people often make decisions on behalf of concrete others rather than imaginary or abstract others in the real world.

Therefore, using a concrete target to indicate others, the present research investigated the influence of social distance on risk preferences in both gain and loss situations. Based on previous findings, we predicted that social distance will have an effect on risk preferences in both gain and loss situations via loss aversion. Specifically, we hypothesized that people may be more risk averse in the gain situation when making decisions for proximal targets than distal targets, but would be more risk seeking in the loss situation when making decisions for proximal targets compared to distal targets.

We conducted two studies to test our hypothesis. In Study 1, we manipulated social distance by asking the participants to make decisions for themselves and for a stranger. In Study 2, we manipulated social distance by asking the participants to make decisions for themselves, a close friend, and a stranger. We also explored whether loss aversion might mediate the relationship between social distance and decisions made for the three types of target.

## Study 1

### Methods

#### Participants and Design

Sixty-one undergraduate students participated in Study 1. Data from three participants were excluded from the analyses because they doubted that the decisions for the other person were real on the post-experiment self-report questionnaire. In addition, data from one participant were also excluded from the analyses due to excessive risk seeking. This participant chose only risky options for all trials in two of the four conditions (self-gain, self-loss, other-gain, and other-loss). We therefore used the data from the remaining 57 participants (31 females, age range 18 to 26 years, mean ± SD = 21.72 ± 2.56 years). This study was approved by the Research Ethics Committee of East China Normal University, and the written informed consent was obtained from all participants involved in the study.

A 2 (social distance: self or other) × 2 (decision situation: gain or loss) within-subjects design was conducted in study 1.

#### Experimental Task and Procedure

We adopted a modified version of the cups task (Weller et al., 2007), which consists of gain and loss domains. Gain domain trials involved the choice between a sure gain of ¥5 (RMB 5) and a designated probability (0.20, 0.25, 0.33, or 0.50) of a larger gain (¥25, ¥20, ¥15, or ¥10) or no gain. Loss domain trials involved the choice between an option that provides a sure loss of ¥5, and another option that provides a designated probability of a larger loss (¥25, ¥20, ¥15, or ¥10) or no loss. In the present study, we only used certain combinations where the levels of probability and magnitude produce equal expected value for the risky and sure options, which offer an ideal measurement of individuals’ risk preferences (Xue et al., 2009). These are the following combinations: 0.20 × 25, 0.25 × 20, 0.33 × 15, and 0.50 × 10, for both gain and loss domain trials.

**FIGURE 1:**
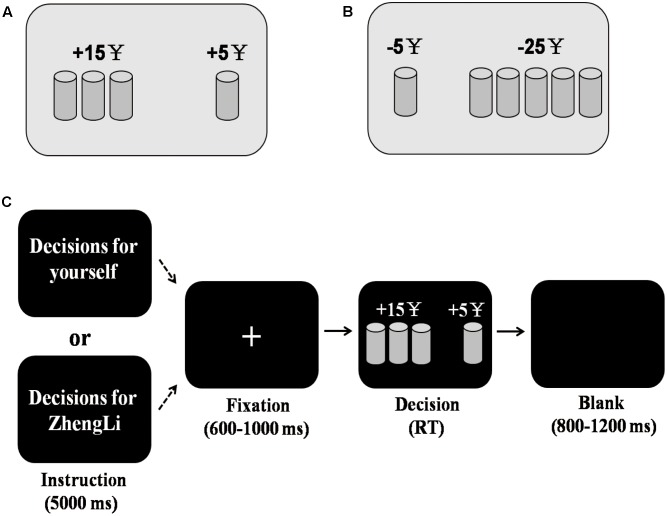
Illustration of the cups task and experimental procedure. **(A)** A gain situation. The risky option was presented on the left side of the screen, with a 0.33 chance of gaining ¥15 or a 0.67 possibility of gaining nothing. The sure option was presented on the right side of the screen, with a certainty of gaining ¥5. **(B)** A loss situation. The risky option was presented on the right side of the screen, with a 0.20 probability of losing ¥25 or a 0.80 probability of losing nothing. The sure option was presented on the left side of the screen, with a certainty of losing ¥5. **(C)** Experimental procedure. In each block, an instruction was presented only at the first trial indicating whether the subsequent series of decisions were made for oneself or for another person (i.e., ZhengLi). During the decision phase, the gain and loss domain trials were presented in a pseudo-random order within each block.

In each trial, a cup was presented on one side of a screen. This side was identified as the sure side where ¥5 was gained (gain domain, **Figure 1A**) or lost (loss domain, **Figure 1B**). The other side of the screen was identified as the risky side where an array of two, three, four, or five cups was presented, and the choice of one cup leads to a specified amount of money being gained or lost, whereas selection of the other cups leads to no gain or no loss. For a risky option, whether the choice of one cup caused a non-zero outcome was determined by a random process where *p* is equal to 1 divided by the number of cups [*p* = 1/(number of cups); Levin et al., 2007; Weller et al., 2007]. As in the previous study (Xue et al., 2009), participants were not required to select a specific cup in the risky option, and they were only required to choose between a risky option and a sure option, which makes the task easier to perform.

To minimize potential effects of gender on decision propensities under risk, a neutral name (“ZhengLi”; in Chinese, both males and females can have this name) was adopted to indicate the stranger. This name was chosen based on a pilot study conducted on 25 additional participants (13 females, age range 22 to 27 years, mean ± SD = 24.32 ± 1.41 years). Participants were informed that the same-sex stranger had been randomly selected from among the participants of another experiment, and that they would never meet.

The formal experiment consisted of four blocks (two blocks for each decision target). Presentation order of these blocks was counterbalanced across participants. At the beginning of each block, an instruction indicating whether it was a self (decision-for-self) or stranger (decision-for-stranger) block was shown to the participants for 5000 ms (**Figure 1C**). Each trial started with the presentation of a central fixation point with a duration that varied randomly from 600 to 1000 ms. Subsequently, a risky and sure option were simultaneously presented on the screen, and participants were instructed to press the number key “3” with their right index finger when they choose the option displayed on the left part of the screen, or to press the number key “4” with their right middle finger when they choose the option displayed on the right part of the screen. Two (risky and sure) options were randomly positioned to the left or right of the screen in every trial. The alternatives remained on the screen until the participants made a choice. After the participants’ response, the trial ended with a blank screen whose presentation duration ranged from 800 to 1200 ms. Notably, the outcome of the choice was not revealed to the participants in each trial, to exclude potential effects of learning from reward feedback (Suzuki et al., 2016). Within each block, 40 trials were presented in a pseudo-random order in which no more than three successive trials were from the same decision situation, consisting of five repetitions for each of the eight combinations (four combinations for each of gain and loss). In total, each participant performed 160 trials.

Participants were given a ¥30 (about United States $4.50) initial endowment before the experiment began so that they could pay any eventual losses at the end of the experiment. They were informed that the computer would randomly select one trial from the self trials at the end of the experiment, and the outcome of the selected trial would be added to or subtracted from the initial endowment, and a payment would be implemented depending on their actual choice. Because participants did not know which trial would be selected, they should have treated every trial independently (De Martino et al., 2010). In addition, participants received a show-up fee of ¥20 (about United States $3).

Before the formal experiment, each participant performed eight practice trials to familiarize themselves with the experimental task. After the experimental task, participants answered a yes/no question: “Do you strongly doubt that the decisions for the stranger during the task were real ?” (used for the exclusion of participants; see *Method, Participants and design*).

### Results and Discussion

A 2 (social distance: self, other) × 2 (decision situation: gain, loss) within- subjects repeated measures ANOVA was conducted on risk rate (i.e., proportion of times that the participants chose the risky option)^1^. The main effect of social distance was significant, *F*(1,56) = 6.85, *p* = 0.011, $\eta_{p}^{2}$ = 0.11, the risk rate was significantly higher when making decisions for the self (*M* = 0.53, *SD* = 0.11) than for others (*M* = 0.51, *SD* = 0.11). A significant main effect of decision situation was also observed, *F*(1,56) = 20.47, *p* < 0.001, $\eta_{p}^{2}$ = 0.27. The risk rate in gain situations (*M* = 0.44, *SD* = 0.13) was significantly lower than in loss situations (*M* = 0.59, *SD* = 0.19).

**FIGURE 2:**
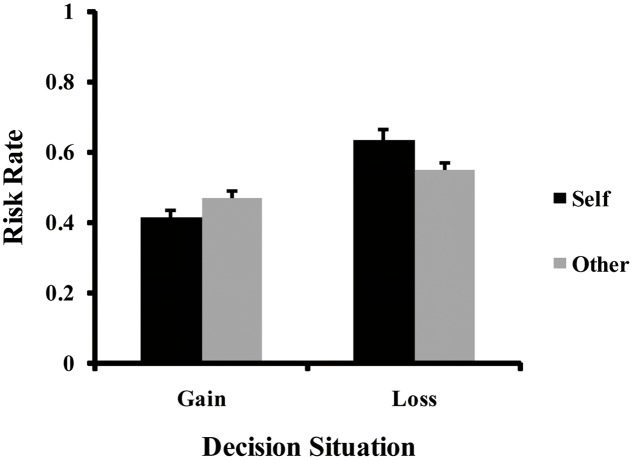
Significant interaction of social distance × decision situation for the risk rate. Error bars indicate standard error.

More interestingly, a significant interaction between social distance and decision situation was observed (see **Figure 2**), *F*(1,56) = 18.42, *p* < 0.001, $\eta_{p}^{2}$ = 0.25. Follow-up simple effects analyses revealed the following. In gain situations, the risk rate was significantly lower when making decisions for the self (*M* = 0.42, *SD* = 0.14) than for others (*M* = 0.47, *SD* = 0.15), *F*(1,56) = 8.94, *p* = 0.004, $\eta_{p}^{2}$ = 0.14. However, the risk rate was significantly higher when making decisions for the self (*M* = 0.64, *SD* = 0.22) than for others (*M* = 0.55, *SD* = 0.18) in loss situations, *F*(1,56) = 23.45, *p* < 0.001, $\eta_{p}^{2}$ = 0.30.

By manipulating social distance, we aimed to investigate the effect of social distance on risk preferences in both gain and loss situations. In line with our hypothesis, people were more risk averse in gain situations when making decisions for themselves than for a stranger, and were more risk seeking in loss situations. Our findings indicated that social distance between decision makers and targets has a significant effect on risk preferences in both gain and loss frames, and moreover, this effect is stronger in loss frames than in gain frames. Yet, in real-world scenarios it is more typical that people make decisions for a close friend as opposed to a complete stranger (Braams et al., 2014; Yu et al., 2015). Furthermore, important decisions are often made for a close friend (Wu et al., 2011). Does it also make a difference whether a decision is made for oneself, a close friend, or a stranger? We next investigated individual risk preferences in both gain and loss situations when making decisions for oneself, a close friend, and a stranger in Study 2.

## Study 2

The findings from Study 1 indicated that social distance exerts a strong influence on decision makers’ risk preferences in both gain and loss situations. In Study 2, we firstly sought to replicate and extend the findings of Study 1 to provide further evidence for the relationship between social distance and risk preferences. Secondly, we investigated whether the loss aversion induced by potential decision outcomes is responsible for the present findings.

### Methods

#### Participants and Design

Ninety-five undergraduate students participated in Study 2. Data from eleven participants were excluded from the analyses because they doubted that the decisions made for their friends or another person were real on the post-experiment self-report questionnaire. Additionally, data from two participants were also excluded from the analyses because of excessive risk seeking. These two participants chose only risky options for all trials in two of the six conditions (self-gain, self-loss, friend-gain, friend-loss, stranger-gain, and stranger-loss). The remaining eighty-two participants (44 females, age range 18 to 26 years, mean ± SD = 20.77 ± 2.48 years) were used in the data analysis. This study was approved by the Research Ethics Committee of East China Normal University, and the written informed consent was obtained from all participants involved in the study.

A 3 (social distance: self, friend or stranger) × 2 (decision situation: gain or loss) within-subjects design was conducted in Study 2.

#### Experimental Task and Procedure

Study 2 used the same task as Study 1. Participants were first instructed to write down the name of a same-sex close friend (Leng and Zhou, 2010, 2014; Wu et al., 2011), and this person’s telephone and wechat numbers. We assured participants that this information would only be used to make a reward payment. As in Study 1, a neutral name (“ZhengLi”) was adopted to indicate the stranger. Participants were also informed that the same-sex stranger had been randomly selected from among the participants of another experiment, and that they would never meet.

The formal experiment consisted of six blocks (two blocks for every target). Presentation order of these blocks was counterbalanced across participants. At the beginning of each block, an instruction indicating a self, friend, or stranger block was shown to the participants for 5000 ms. Within each block, 40 trials were presented in a pseudo-random order in which no more than three successive trials were from the same decision situation, consisting of five repetitions for each of the eight combinations (four combinations for each of gain and loss). The experimental procedure for every trial was the same as in Study 1. In total, each participant performed 240 trials. The method of reward payment was also the same as in Study 1. Reward payments regarding decisions for their friends were administered in the same fashion as those for decisions for themselves. Participants were given a ¥30 initial endowment before the experiment began so that they could pay any eventual losses regarding decisions for their friends at the end of the experiment. They were informed that the computer would randomly select one trial from the friend trials at the end of the experiment, and the outcome of the selected trial would be added to or subtracted from the initial endowment, and a payment would be made depending on their actual choice for their friends during the experiment.

Before the formal experiment, each participant performed 12 practice trials to familiarize themselves with the experimental task. After the experimental task, participants answered two yes/no questions: “Do you strongly doubt that the decisions for your friend during the task were real?” and “Do you strongly doubt that the decisions for the stranger during the task were real?” (used for the exclusion of participants; see *Method, Participants and design*).

After the experimental task, participants were also asked to complete a 5-point scale to rate their emotional response to the potential outcomes. Specifically, participants were required to evaluate how they felt if their choices lead to themselves’/their friends’/the stranger’s gains/losses in the gain/loss situation (1 = “very unhappy” to 5 = “very happy”).

Participants were subsequently required to complete the Chinese version of the inclusion of other in the self (IOS; Aron et al., 1992) scale. The IOS measures, with different degrees of overlap between two circles (ranging from 1 to 7; higher scores representing more inclusion), described the perceived closeness between the participants themselves and their friends as well as the stranger. As in previous studies (Wu et al., 2011; Yu et al., 2015), the IOS scale was used to check the manipulation of social distance in the present study.

### Results and Discussion

#### Manipulation Check of Social Distance

Eighty-two participants’ self-reports regarding the IOS revealed that they generally had close relationships with their friends. Participants’ self-reports regarding IOS demonstrated that they had closer relationships with their friends (*M* = 6.22, *SD* = 0.61) than with the stranger (*M* = 1.41, *SD* = 0.50), *t*(81) = 45.87, *p* < 0.001.

#### Risk Rate

A 3 (social distance: self, friend, stranger) × 2 (decision situation: gain, loss) within-subjects ANOVA with repeated measures was conducted on risk rate^2^. The main effect of social distance was significant, *F*(2,162) = 12.00, *p* < 0.001, $\eta_{p}^{2}$ = 0.13. Pairwise comparisons revealed that the risk rate was significantly higher when making decisions for the self (*M* = 0.54, *SD* = 0.10) than for friend (*M* = 0.52, *SD* = 0.09, *p* = 0.035) and stranger (*M* = 0.49, *SD* = 0.14, *p* < 0.001). Moreover, the risk rate was significantly higher when making decisions for friend than for stranger (*p* = 0.033). The main effect of decision situation was also significant, *F*(1,81) = 58.12, *p* < 0.001, $\eta_{p}^{2}$ = 0.42. The risk rate in gain situations (*M* = 0.45, *SD* = 0.08) was significantly lower than that in loss situations (*M* = 0.59, *SD* = 0.15).

**FIGURE 3:**
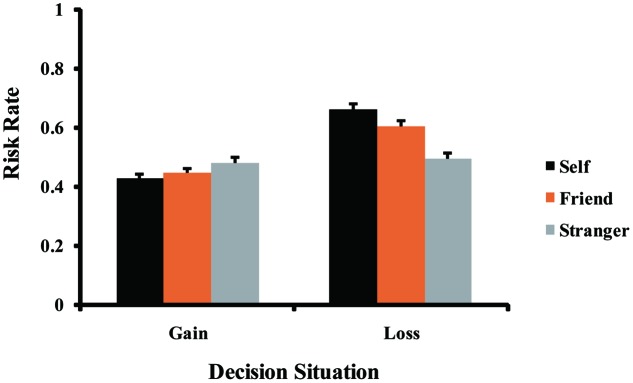
Significant interaction of social distance × decision situation for the risk rate. Error bars indicate standard error.

More interestingly, a significant interaction between social distance and decision situation was observed (see **Figure 3**), *F*(2,162) = 35.85, *p* < 0.001, $\eta_{p}^{2}$ = 0.31. Follow-up simple effects analyses revealed the following. In gain situations, the risk rate difference between different social distance was significant, *F*(2,162) = 4.50, *p* = 0.022. $\eta_{p}^{2}$ = 0.05. Specifically, the risk rate was significantly lower when making decisions for the self (*M* = 0.43, *SD* = 0.12) than for stranger (*M* = 0.48, *SD* = 0.14, *p* = 0.037). However, the risk rate did not differ significantly between making decisions for the self and for friend (*M* = 0.45, *SD* = 0.12, *p* = 0.452), and was also not significant between making decisions for friend and for stranger (*p* = 0.239). In loss situations, the risk rate difference was also significant across the social distances, *F*(2,162) = 51.23, *p* < 0.001, $\eta_{p}^{2}$ = 0.39. More specifically, risk rate was significantly higher when making decisions for the self (*M* = 0.66, *SD* = 0.20) than for friend (*M* = 0.60, *SD* = 0.17, *p* = 0.001) and for stranger (*M* = 0.50, *SD* = 0.17, *p* < 0.001). Moreover, risk rate was also significantly higher when making decisions for friend than for stranger (*p* < 0.001).

#### Mediation Analyses

Of additional interest was whether loss aversion differences between social distances might account for the different pattern of risk preferences. In order to calculate loss aversion values, we first conducted inverse integration on the subjective ratings of unhappiness toward losses. Loss aversion can be indicated by the differences between unhappiness ratings of losses and happiness ratings of gains in the present study. We used techniques suggested by Judd et al. (2001) for testing mediation in within-subject designs.

The first criterion for mediation is that the independent variable (i.e., social distance) must affect the dependent variables (i.e., the risk rate difference between loss and gain situations). It was met by an analysis showing that the risk rate difference between loss and gain situations in different social distance conditions was significant, *F*(2,162) = 35.85, *p* < 0.001, $\eta_{p}^{2}$ = 0.31. The risk rate difference between loss and gain situations was significantly larger when making decisions for the self (*M* = 0.23, *SD* = 0.26) than for friend (*M* = 0.16, *SD* = 0.22, *p* = 0.005) and for stranger (*M* = 0.01, *SD* = 0.13, *p* < 0.001). Moreover, it was also significantly larger when making decisions for friend than for stranger (*p* < 0.001).

The second criterion for mediation is that the independent variable must affect the mediating variables (i.e., loss aversion). This was also met by an analysis indicating that loss aversion differences were significant across social distance conditions, *F*(2,162) = 14.08, *p* < 0.001, $\eta_{p}^{2}$ = 0.15. Loss aversion was significantly stronger when making decisions for the self (*M* = 0.56, *SD* = 1.29) than for friend (*M* = 0.30, *SD* = 1.12, *p* = 0.022) or stranger (*M* = -0.09, *SD* = 0.74, *p* < 0.001). Moreover, it was also significantly stronger when making decisions for friend than for stranger (*p* = 0.01).

For the third criterion, mediation was tested by computing the risk rate differences between each two targets for the difference of loss minus gain situation, respectively, and then regressing this risk rate difference on two predictors: the sum of each participant’s loss aversion scores for each two decision targets, and its difference between the two decision targets (see Judd et al., 2001). As shown in **Table 1**, loss aversion completely mediated the effects of social distance (self vs. friend) on the risk rate difference between loss and gain situations (assuming that the sum variable was centered), and partially mediated the effects of social distance (self vs. stranger and friend vs. stranger) on the risk rate difference between loss and gain situations.

**Table 1:** Loss aversion mediated the effect of social distance on risk rate difference between loss and gain situations.

	Dependent variable *Y* in the model											
	Self vs. friend				Self vs. stranger				Friend vs. stranger			
Predictor	*B*	*SE*	*t*	*p*	*B*	*SE*	*t*	*p*	*B*	*SE*	*t*	*p*
*d*_0_	-0.03	0.02	-1.63	0.106	-0.13	0.02	-5.39	0.000	-0.09	0.02	-4.49	0.000
*X*_S_ (*d*_1_)	0.001	0.01	0.08	0.934	-0.04	0.02	-2.32	0.023	-0.04	0.01	-2.71	0.008
*X*_D_ (*d*_2_)	0.19	0.02	11.20	0.000	0.11	0.02	5.67	0.000	0.13	0.02	7.66	0.000

*Following Judd et al. (2001), mediation of effects of a within subjects factor on a dependent variable *Y* by a mediator *X* was tested with the equation *Y*_*D*_ = *d*_*0*_ + *d*_*1*_*X*_*S*_ + *d*_*2*_*X*_*D*_, where *Y*_*D*_ is the difference between the social distance conditions in *Y*, *X*_*S*_ is the sum of *X* in both conditions, and *X*_*D*_ is the difference between social distance conditions in *X*. In all displayed regressions, the tested mediator *X* was loss aversion*.

In summary, Study 2 further supports the findings of Study 1, indicating that people were more risk averse in gain situations when making decisions for themselves than for a stranger, and were equally risk averse for themselves and their friends, and were also equally risk averse for their friends and a stranger. However, people were more risk seeking in loss situations when making decisions for themselves than for their friends which, in turn, were also more risk seeking than for a stranger. These findings once again showed that social distance significantly influences decision makers’ risk preferences in both gain and loss situations, and moreover, that the effect is stronger in loss frames than in gain frames. In addition, Study 2 demonstrated that the loss aversion induced by potential decision outcomes was responsible for these findings. Participants experienced more loss aversion associated with certain losses derived from a sure prospect in loss situations, and thus, they were more risk seeking when making decisions for themselves than for their friends or for a stranger. On the other hand, they also experienced more loss aversion associated with potential losses derived from a risky prospect in gain situations, which led to more risk-averse behaviors when making decisions for themselves than for a stranger.

## General Discussion

The present research revealed that people were more risk averse in gain situations when making decisions for themselves than for a stranger (Studies 1 and 2), and were equally risk averse for themselves and their friends under such conditions (Study 2). However, people were more risk seeking in loss situations when making decisions for themselves than for their friends, and also more risk seeking for their friends than stranger. These findings indicated that social distance has a robust effect on decision makers’ risk preferences in both gain and loss situations. Moreover, the effect of social distance is stronger in loss than gain situations. Furthermore, our research revealed that the loss aversion induced by potential outcomes was responsible for the effects of social distance, which indicated that self-other differences in decision making under risk may be driven primarily by emotion.

### Social Distance and Risk Preference

Our research extends Ziegler and Tunney’ (2015) study by showing that the effect of social distance is stronger in loss situations than in gain situations, whereas Ziegler and Tunney (2015) showed that this effect was slightly stronger in gain situations than in loss situations where this effect was only approaches significance. Additionally, in the gain frame, our research replicates and extends the two previous studies (Raue et al., 2015; Sun et al., 2016), however, in the loss frame, stands in contrast to them that have reported there was no significant effect of social distance on risk preference in loss frames. This inconsistency between the present findings and these earlier results may be due to either differences in the “others” manipulation or the reward payment used in the studies. The “others” used in the present study were concrete targets both in Study 1 (a concrete stranger) and Study 2 (a concrete friend and stranger), whereas previous studies have used an imaginary or abstract target to indicate the “other.” Crucially, individuals’ risk preference is driven by their emotional reactions to risk (Hsee and Weber, 1997; Loewenstein et al., 2001). When making decisions for a concrete “other,” individuals base their decision partly on their own emotional reactions toward risk, and how much individuals base their decision on their own emotional reactions depends on how concrete the other is. When making decisions for an abstract “other,” people likely find it more difficult to imagine how the other feels about risk, which indicates a lack of particular feelings and contributes to risk neutrality when making decisions for the “other” (Hsee and Weber, 1997). If this account is accurate, concreteness of target would be expected to have a major impact on decision makers’ risk preferences.

Of course, use of different reward payment could also impact the pattern of findings obtained. Participants in previous studies received the same gifts (Sun et al., 2016), the same course credit (Raue et al., 2015), or a financial reward that comprised outcomes for all choices in the self condition and in the previous but one participant condition (Ziegler and Tunney, 2015). Rewards that do not depend on task performance or that are partly dependent on the choices from others maybe contribute to similar risk-seeking behaviors regardless of decision-making target. In the present research, participants were given an initial endowment before the experiment began, and were told that one trial in the self condition would be randomly selected at the end of the experiment and a payment made depending on their actual choice. This design could increase participants’ emotional engagement, more importantly, because participants did not know which trial would be selected, and thus they should treat every trial independently (De Martino et al., 2010; Suzuki et al., 2016).

In addition, Stone et al. (2002), Stone and Allgaier (2008) found that there was no difference in risk-taking behavior between decisions for the self and friend in monetary gain situations. They also showed that, in low-impact relationship situations (e.g., introducing him- or herself at a party), people were more risk-averse when making decisions for the self than for friends or a typical student, and there was no difference between making decisions for friends and for a typical student, whereas no self-other differences have been observed in high-impact relationship situations (e.g., eloping with someone) (Stone and Allgaier, 2008). In addition, people were more risk-seeking when making decisions for themselves than for their friends in physical safety situations (e.g., taking diet pills), but were more risk-averse in romantic relationship situations (e.g., asking an attractive person to dance) (Stone et al., 2013). Stone and Allgaier (2008) suggested that decision making for others is heavily dependent on the social value placed on risk, which they termed the “social values analysis.” Risk taking is valued in low-impact relationship situations but is not valued in high-impact relationship and monetary situations (Stone and Allgaier, 2008), whereas risk aversion is valued in physical safety situations (Stone et al., 2013). Thus, our findings are basically consistent with the social values analysis. Individuals tend to make more risk-seeking decisions for stranger than for themselves in gain situations, because risk taking is more valued under such circumstances. However, individuals tend to be more risk-averse when making decisions for their friends and stranger than for themselves in loss situations, because risk aversion is more valued in these scenarios.

### Other Mechanisms for Social Distance Affects Risk Preference

The present research investigated loss aversion as a potential mediator of the influence of social distance on risk preference. However, other mechanisms may also exist. Construal level represents one such mechanism. According to the construal level theory (Trope et al., 2007; Trope and Liberman, 2010), psychological distance (in terms of social distance, space, time, and hypotheticality) has an impact on human decision making by activating a certain level of construal. Psychological closeness is associated with low-level, concrete construals, whereas psychological distance is associated with high-level, abstract construals. Use of low-level, concrete construals leads individuals to focus more on the feasibility of an action (e.g., the possibility of a gain), whereas the use of high-level, abstract construals shifts more focus onto desirability (e.g., attractiveness of a gain). Therefore, people might be more risk averse in gain situations and more risk seeking in loss situations when making decisions for close social distance targets due to activating a low-level construal, and it maybe go just the opposite for distant social distance targets.

Another possible explanation for the observed effect of social distance on risk preference is the reference point. The current state of individuals is represented based on a reference point, and gains and losses are thus weighed relative to this point (Epley et al., 2004; Epley and Gilovich, 2006). However, decision-makers have no reference point for the current state of others, and they would resort more to risk neutrality when making decisions for distant social targets.

### Theoretical Contributions and Practical Implications

Our findings contribute to prospect theory (Kahneman and Tversky, 1979) by showing that risk preference varies based on social distance. In the close social distance condition, people tend to be risk averse in gain frames and risk seeking in loss frames. However, greater social distance makes people become more risk-neutral in both gain and loss frames. These findings indicate that the prospect theory is more applicable to explain decisions for close social distance targets rather than distant targets. Additionally, the present research revealed that social distance has a stronger effect on decision makers’ risk preference in loss frames than in gain frames, and that loss aversion is responsible for this effect of social distance. These findings shed new light on self-other decision-making differences in risk preference.

Our findings also have potential practical implications. For instance, asset managers tend to be more rational (i.e., risk-neutral) when making decisions for their investors than for themselves. In addition, it is best for physicians not to make medical decisions when the decision targets are themselves or their friends, because the close social distance may hamper professional judgment. Our findings also have potential implications for understanding substance abuse and addictive behaviors. In the real world, people often face a variety of losses. Our findings imply that the more susceptible to losses could potentially contribute to increases in behaviors resulting in maladaptive outcomes such as pathological gambling, drug-taking, and substance abuse.

### Limitations and Directions for Future Research

We acknowledge several potential caveats to this research. Although we have revealed significant effects of social distance on risk preferences in both gain and loss situations, potential differences based on subjective value or some aspect of subjective value such as expected value, reward probability, reward magnitude, and stochasticity, have not been clearly identified. Thus, future research is needed to explore the computational mechanisms underlying the differences using model fitting. For example, researchers can estimate the risk preference parameter based on cumulative prospect theory (CPT, Tversky and Kahneman, 1992) by fitting a model to the participant’s choice data (i.e., estimating the parameter values that maximize the likelihood function). Such research could try to pinpoint what aspects of the choice process are altered and may shed further light on the mechanisms underlying the effect of social distance on risk preference.

## Conclusion

Across two experiments, social distance and decision situation interacted to affect individuals’ risk preference. People were more risk averse in gain situations when making decisions for themselves than for a stranger but were equally risk averse for themselves and for their friends. They were more risk seeking in loss situations when making decisions for themselves than for their friends which, in turn, were more risk seeking than for a stranger. Moreover, the effect of social distance on risk preference was stronger in loss situations than in gain situations, and loss aversion induced by potential outcomes was responsible for the effects of social distance. Taken together, our findings indicate that social distance dramatically affects decision makers’ risk preferences in both gain and loss situations via perceived loss aversion.

## Author Contributions

XZ, YoL, and YiL designed this research. XZ, XC, and XS performed this research. XZ, XC, YiL, and XS analyzed the data and joined in the interpretation of data. XZ, YoL, YiL, and XC wrote this paper.

## Conflict of Interest Statement

The authors declare that the research was conducted in the absence of any commercial or financial relationships that could be construed as a potential conflict of interest.

## Footnotes

1 The data without any participants excluded were also analyzed. In this case, the main effects of social distance and decision situation were less robust, whereas the interaction between social distance and decision situation was enhanced when every participant was included. The simple effects for gain situations were also more robust, whereas these effects were weaker for loss situations. More detailed results with no participants excluded are reported in the **Supplementary Materials**.

2 The data without any participants excluded were also analyzed. In this case, the main effects of social distance and decision situation were less robust, and the interaction between social distance and decision situation was also less robust when every participant was included. The simple effects for gain situations were more robust, whereas the simple effects were weaker for loss situations. More detailed results with no participants excluded are reported in the **Supplementary Materials**.

## Supplementary Material

FILE S1 Post-experiment Questionnaire, a 5-point scale to rate subjects’ emotion response towards potential outcomes, the inclusion of other in the self scale (IOS), and results regarding every participant was included.
