# Supplementary material for: Decisions for Others Are Less Risk-Averse in the Gain Frame and Less Risk-Seeking in the Loss Frame Than Decisions for the Self
Source: Front Psychol. 2017 Sep 15;8:1601. doi: 10.3389/fpsyg.2017.01601 (PMC5605664; doi:10.3389/fpsyg.2017.01601)
Supplement: FILE S1 — Post-experiment Questionnaire, a 5-point scale to rate subjects’ emotion response towards potential outcomes, the inclusion of other in the self scale (IOS), and results regarding every participant was included. [file Presentation_1.PDF]

Post-experiment Questionnaire.

After the experimental task of Study 1, participants were asked to answer a yes/no question:

Do you strongly doubt that the decisions for the stranger during the task were real?

A: Yes B: No

After the experimental task of Study 2, participants were asked to answer two yes/no questions:

1. *Do you strongly doubt that the decisions for your friend during the task were real*?

   A: Yes B: No

2. *Do you strongly doubt that the decisions for the stranger during the task were real*?

   A: Yes B: No

5-point scale to rate subjects' emotion response towards potential outcomes

After the experimental task of Study 2, participants were asked to rate:

1. How do you feel if your choices leading to yourselves gains in the gain situation?

   1- very unhappy 2- unhappy 3- neutral 4- happy 5- very happy

2. How do you feel if your choices leading to your friend gains in the gain situation?

   1- very unhappy 2- unhappy 3- neutral 4- happy 5- very happy

3. How do you feel if your choices leading to the stranger gains in the gain situation?

   1- very unhappy 2- unhappy 3- neutral 4- happy 5- very happy

4. How do you feel if your choices leading to yourselves losses in the loss situation?

   1- very unhappy 2- unhappy 3- neutral 4- happy 5- very happy

5. How do you feel if your choices leading to your friend losses in the loss situation?

   1- very unhappy 2- unhappy 3- neutral 4- happy 5- very happy

6. How do you feel if your choices leading to the stranger losses in the loss situation?

   1- very unhappy 2- unhappy 3- neutral 4- happy 5- very happy

The inclusion of other in the self scale (IOS).

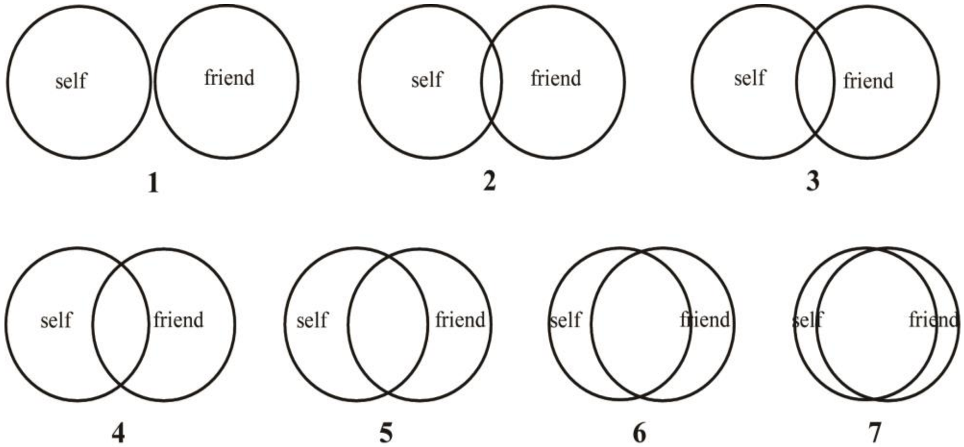

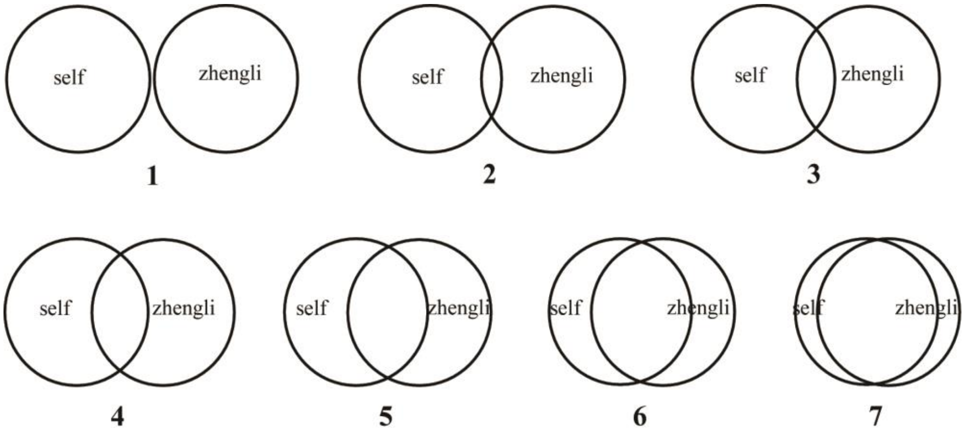

Results regarding every participant was included

Study1

A 2 (social distance: self, other) × 2 (decision situation: gain, loss) within- subjects ANOVA with repeated measures was conducted on risk rate. The main effect of social distance was not significant, $F(1, 60) = 2.62$, $p = 0.111$, $\eta_p^2 = 0.04$. A significant main effect of decision situation was observed, $F(1, 60) = 20.07$, $p < 0.001$, $\eta_p^2 = 0.25$. The risk rate in gain situations ($M = 0.46$, $SD = 0.16$) was significantly lower than in loss situations ($M = 0.61$, $SD = 0.20$).

More interestingly, a significant interaction between social distance and decision situation was observed, $F(1, 60) = 22.21$, $p < 0.001$, $\eta_p^2 = 0.27$. Follow-up simple effects analyses revealed the following effects. In gain situations, the risk rate was significantly lower when making decisions for the self ($M = 0.43$, $SD = 0.16$) than for others ($M = 0.49$, $SD = 0.17$), $F(1, 60) = 12.24$, $p = 0.001$, $\eta_p^2 = 0.17$. However, the risk rate was significantly higher when making decisions for the self ($M = 0.65$, $SD = 0.22$) than for others ($M = 0.56$, $SD = 0.20$) in loss situations, $F(1, 60) = 21.41$, $p < 0.001$, $\eta_p^2 = 0.26$.

Study2

A 3 (social distance: self, friend, stranger) × 2 (decision situation: gain, loss) within-subjects ANOVA with repeated measures was conducted on risk rate. The main effect of social distance was significant, $F(2, 188) = 6.64$, $p = 0.005$, $\eta_p^2 = 0.07$. Pairwise comparisons revealed that the risk rate was significantly higher when making decisions for the self ($M = 0.55$, $SD = 0.11$) than for stranger ($M = 0.51$, $SD = 0.16$, $p = 0.007$).

However, the risk rate was not significant between making decisions for the self and for friend (M = 0.54, SD = 0.11, p = 0.442), and the risk rate was also not significant between making decisions for friend and for stranger (p = 0.087). The main effect of decision situation was also significant, $F(1, 94) = 61.24$, $p < 0.001$, $\eta_p^2 = 0.39$. The risk rate in gain situations (M = 0.46, SD = 0.09) was significantly lower than in loss situations (M = 0.60, SD = 0.18).

More interestingly, a significant interaction between social distance and decision situation was observed, $F(2, 188) = 32.78$, $p < 0.001$, $\eta_p^2 = 0.26$. Follow-up simple effects analyses revealed the following effects. In gain situations, the risk rate difference between different social distance was significant, $F(2, 188) = 5.67$, $p = 0.011$, $\eta_p^2 = 0.06$. Specifically, the risk rate was significantly lower when making decisions for the self (M = 0.42, SD = 0.13) than for friend (M = 0.46, SD = 0.14, p = 0.009) and for stranger (M = 0.49, SD = 0.17, p = 0.018). However, the risk rate did not differ significantly between making decisions for friend and for stranger (p = 0.464). In loss situations, the risk rate difference between different social distance was also significant, $F(2, 188) = 48.27$, $p < 0.001$, $\eta_p^2 = 0.34$. More specifically, the risk rate was significantly higher when making decisions for the self (M = 0.67, SD = 0.21) than for friend (M = 0.61, SD = 0.18, $p < 0.001$) and for stranger (M = 0.52, SD = 0.19, $p < 0.001$). Moreover, it was also significantly higher when making decisions for friend than for stranger ($p < 0.001$).
